# Formin1 Mediates the Induction of Dendritogenesis and Synaptogenesis by Neurogenin3 in Mouse Hippocampal Neurons

**DOI:** 10.1371/journal.pone.0021825

**Published:** 2011-07-19

**Authors:** Julia Simon-Areces, Ana Dopazo, Markus Dettenhofer, Alfredo Rodriguez-Tebar, Luis Miguel Garcia-Segura, Maria-Angeles Arevalo

**Affiliations:** 1 Laboratory of Neuroactive Steroids, Instituto Cajal, Consejo Superior de Investigaciones Cientificas (CSIC), Madrid, Spain; 2 Genomics Unit, Centro Nacional de Investigaciones Cardiovasculares (CNIC), Madrid, Spain; 3 Department of Genetics, Harvard Medical School, Boston, Massachusetts, United States of America; 4 Centro Andaluz de Biología Molecular y Medicina Regenerativa/Consejo Superior de Investigaciones Cientificas (CABIMER/CSIC), Seville, Spain; Dalhousie University, Canada

## Abstract

Neurogenin3, a proneural transcription factor controlled by Notch receptor, has been recently shown to regulate dendritogenesis and synaptogenesis in mouse hippocampal neurons. However, little is known about the molecular mechanisms involved in these actions of Ngn3. We have used a microarray analysis to identify Ngn3 regulated genes related with cytoskeleton dynamics. One of such genes is *Fmn1*, whose protein, Formin1, is associated with actin and microtubule cytoskeleton. Overexpression of the *Fmn1* isoform-Ib in cultured mouse hippocampal neurons induced an increase in the number of primary dendrites and in the number of glutamatergic synaptic inputs at 4 days in vitro. The same changes were provoked by overexpression of *Ngn3*. In addition downregulation of *Fmn1* by the use of *Fmn1*-siRNAs impaired such morphological and synaptic changes induced by *Ngn3* overexpression in neurons. These results reveal a previously unknown involvement of Formin1 in dendritogenesis and synaptogenesis and indicate that this protein is a key component of the Ngn3 signaling pathway that controls neuronal differentiation.

## Introduction

Neurogenin3 (Ngn3) is a transcription factor whose expression is negatively controlled by the activity of Notch receptor [1]. Ngn3 is mostly known as a proendocrine factor implicated in the differentiation of precursors of the four endocrine cell lineages in the developing pancreas [2]. In addition, Ngn3 is also involved in the development of neurons and glial cells in the central nervous system. In chick embryos, Ngn3 promotes early retinal neurogenesis [3]. In rodents, Ngn3 is expressed in glial precursors in the developing spinal cord [4] and regulates glial differentiation [5]. Furthermore, Ngn3 participates in the control of neuronal differentiation, regulating dendritogenesis and synaptogenesis in hippocampal neurons [6], [7] two processes that involve a remodeling of actin and microtubule cytoskeleton. In order to identify the molecular mechanisms mediating Ngn3 actions on neuronal development we used microarray technology [8]. Using this approach and focusing on genes related with cytoskeletal reorganization, among those upregulated by Ngn3, we identified *Fmn1*, which encodes for Formin1.

The formin proteins consist of approximately 25 family members, and are widely expressed in eukaryotic cells [9]–[11]. The formins are defined by the presence of the formin homology 2 domain (FH2), which was originally shown to be sufficient for the nucleation of filamentous actin at its barbed-end [12]–[15]. The founding member of the formin family, Formin1 consists of six different mRNA isoforms (Ia, Ib, II, III, IV and V) that are differentially expressed in mammalian tissues [16]–[19]. *Fmn1-IV* is localized to the cytoplasm of fibroblasts and epithelial cells, and is detected at concentrated points along microtubules. Primary cells where *Fmn1-IV* has been genetically disrupted display cell spreading and focal adhesion formation defects [20]. Additionally, when isoform Ib of Formin1 (*Fmn1-Ib*) is exogenously expressed, it is almost exclusively cytoplasmic and specifically localizes to interphase microtubules. This localization is regulated by the peptide encoded by exon 2 of the *Fmn1-Ib* gene and does not depend on the FH2 or other domains. This suggests that independent regions of the Formin1-Ib protein are responsible for its association with the actin and microtubule cytoskeletons [21].

We show here that overexpression of *Fmn1-Ib* produces an increase in the number of primary dendrites in cultured hippocampal neurons and an increase in the number of glutamatergic synaptic inputs. In addition, siRNA mediated downregulation of *Fmn1* impaired the morphological and synaptic changes induced by Ngn3. This suggests that Ngn3 regulates dendritogenesis and synaptogenesis through the actions of Formin1.

## Results

### Microarray analysis

324 genes were found to be differentially expressed between cultured hippocampal neurons overexpressing *Ngn3* and control cultures (overexpressing *GFP*) at false discovery rate (FDR) = 5% after multiple testing corrections to control the FDR across all genes. Among the 324 genes, expression levels of 186 genes are up-regulated and 138 genes are down-regulated by *Ngn3* overexpression (Table S1, Supporting Information).

**Figure 1:**
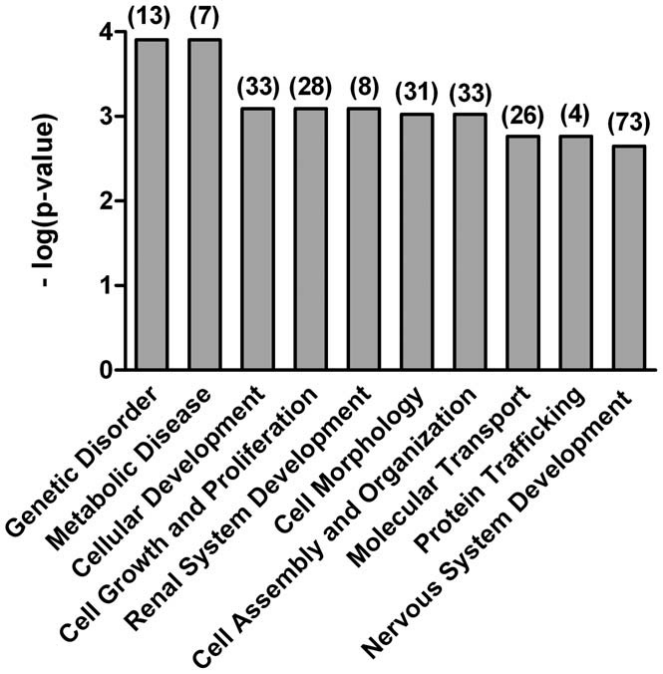
Top ten signaling pathways regulated by Ngn3. For the functional categorization of genes, Fischer's exact test was used to calculate a p value (shown as bars) determining the probability that each biological function assigned to the network is due to chance alone. The number of modified genes for each pathway is shown between brackets.

Then, genes regulated by Ngn3 were organized by function to better understand their profile. The functional characterization of data are presented in Figure 1, which lists the top ten canonical pathways regulated by Ngn3. Genes included in each group of the top ten signaling pathways presented in Figure 1 are listed in Table S2 (Supporting Information).

### Validation of expression profiling results

**Figure 2:**
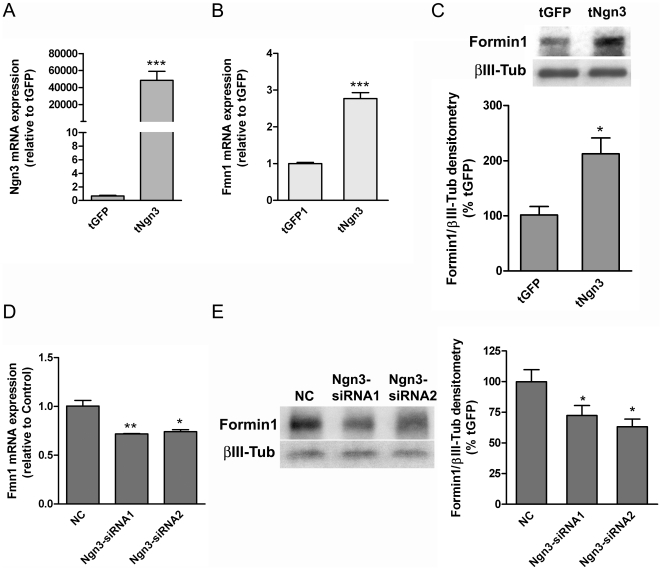
Differential *Fmn1* mRNA and protein expression in neurons overexpressing *GFP* or *Ngn3*. Cultured hippocampal neurons were transducted at 5 DIV with Sindbis virus expressing *Ngn3* or *GFP* and the *Ngn3* and *Fmn1* mRNA expression levels were analyzed in the extracts of both samples. (A, B) Graph represents the relative expression of the *Ngn3* and *Fmn1* gene as determined by real time RT-PCR. (C) Western blots showing the expression levels of Formin1 protein after overexpression of *Ngn3*. Staining for βIII-tubulin was included as loading control. (D) SiRNAs targeted to *Ngn3* or non targeting siRNA (negative control, NC) were nucleofected into parallel cultures using an Amaxa nucleofector with the Mouse Neuron Kit and *Fmn1* mRNA expression was evaluated. (E) Western blots showing the expression levels of Formin1 protein after knocking down of *Ngn3*. Staining for βIII-tubulin was included as loading control. Data show the mean ± SEM from three different experiments. Significance levels were determined using a Student t-test; * p<0.05, ** p<0.01, *** p<0.001.

One of the genes upregulated by Ngn3 is *Fmn1*, which encodes for Formin1. This gene is included in the group of genes associated with Cellular Assembly and Organization (Table S2). Formin1 is involved in nucleation and assembly of actin filaments, processes that have been directly implicated in dendritogenesis [22], [23]. Although *Fmn1* has previously been shown to be expressed in neurons [16], [24], the precise function of Formin1 in the nervous system is unknown. To validate the differential expression of *Fmn1*, detected by microarray analysis, we used real time RT-PCR and Western blot. Hippocampal neuronal cultures were incubated with Sindbis virus expressing either *Ngn3* or *GFP* and analyzed for *Ngn3* and *Fmn1* mRNA and Formin1 protein expression levels in the extracts of both samples. The overexpression of *Ngn3* induced a marked increase of *Ngn3* mRNA levels, as expected (Figure 2A), as well as a significant increase of *Fmn1* mRNA (Figure 2B) and protein (Figure 2C) levels. Furthermore, the downregulation of *Ngn3* gene using siRNA oligonucleotides lead to a significant decrease in *Fmn1* mRNA (Figure 2D) and protein (Figure 2E) levels.

### Overexpression of *Fmn1-Ib* changes the dendritic morphology of hippocampal neurons

**Figure 3:**
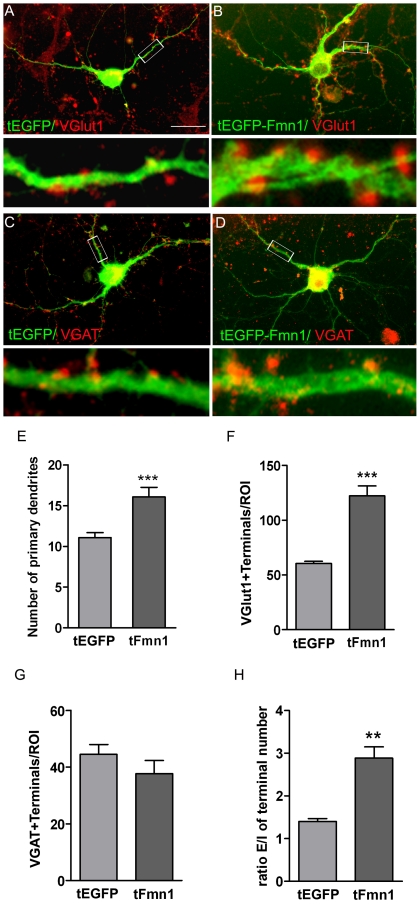
Effects of *Fmn1-Ib* overexpression on the morphology and synaptology of cultured hippocampal neurons. Cells were tranfected at 3 DIV with an *EGFP* (A and C) or *EGFP-Fmn1-Ib* (B and D) expressing *C2* vector and immunostained with antibodies against GFP and VGlut1 or VGAT. (A–D) Representative immunofluorescence images of neurons marked in green for GFP and with its synaptic contacts marked in red. Scale bar, 25 µm. Lower panels show the boxed regions at higher magnification. (E) Number of primary dendrites. (F) Counts of VGlut1 immunoreactive terminals in contact with a neuron within a circular region of interest (ROI) with a diameter of 50 µm and centered in the neuronal soma. (G) Counts of VGAT immunoreactive terminals in contact with a neuron per ROI. Typically 60–75 neurons were evaluated in each condition (n = 3). (H) Ratio of excitatory/inhibitory (E/I) synaptic terminal number. Data are mean ± SEM and significance levels were determined using a Student t-test; ** p<0.01, *** p<0.001 versus EGFP expressing neurons values.

We have previously reported that under conditions of intermediate cell density, overexpression of *Ngn3* stimulates the sprouting of new dendrites in cultured hippocampal neurons [6]. Conversely, addition of NGF to cultures induces the down-regulation of *Ngn3* mRNA levels and hence hippocampal neurons sprout fewer primary and secondary dendrites. Since Ngn3 enhances the expression of *Fmn1*, we decided to test the effects of *Fmn1* overexpression on hippocampal neuronal development. Hippocampal neuronal cultures were transfected with vectors expressing *EGFP* and *EGFP-Fmn1-Ib*. Specimen images of transfected cells are presented in Figure 3, A–D. As expected, the protein encoded by *EGFP-Fmn1-Ib* was not found in the nuclei. The quantification of the results showed that overexpression of *Fmn1-Ib* resulted in a clear stimulation of dendrite initiation (Figure 3E). The number of primary dendrites was increased by 50% versus control levels by 16 h after transfection with *EGFP-Fmn1-Ib*.

### Overexpression of *Fmn1-Ib* increases the number of glutamatergic synaptic terminals without modification of inhibitory terminal numbers

Double immunostaining experiments with neurons expressing *EGFP* alone or *EGFP*-tagged *Fmn1-Ib* were performed to test for a specific change in the number of glutamatergic as opposed to GABAergic synaptic terminals. In all experiments, transfected cells grew in a dense network of neurons, ensuring the availability of a proper synaptic network and excluding differences in synapse number resulting from different cell densities. Glutamatergic and GABAergic terminals were identified with antibodies against the respective vesicular transmitter transporters VGlut1 (Figure 3A and B) and VGAT (Figure 3C and D). It can be seen that neurons responded to the over-expression of *Fmn1* with a clear increase in the number of glutamatergic presynaptic terminals (Figure 3F). In contrast, the number of GABAergic presynaptic terminals was not significantly changed (Figure 3G). This resulted in a significant increase in the ratio of excitatory/inhibitory synaptic inputs (Figure 3H).

### Formin1 mediated the effects of Ngn3 on neuronal morphology and number of synaptic terminals in hippocampal cultures

**Figure 4:**
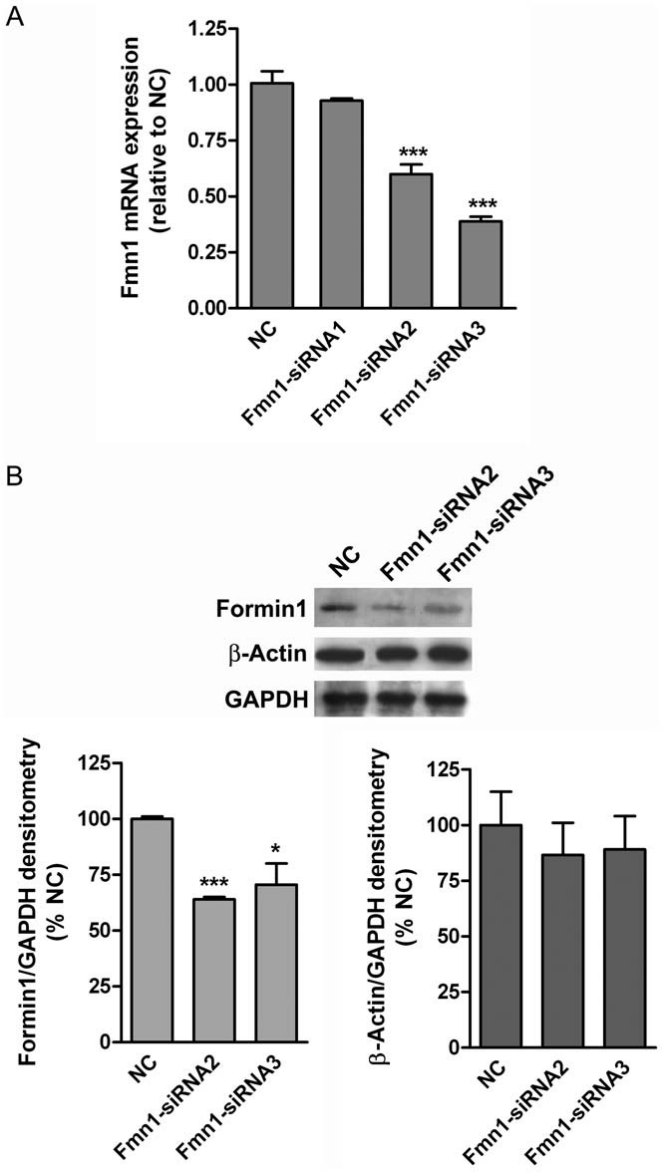
Effects of siRNAs on the levels of *Fmn1* and *β-Actin* mRNA expression. Dissociated E17 hippocampal cells were nucleofected with siRNAs targeting *Fmn1* (siRNA1, siRNA2 and siRNA3) and non targeting siRNA (negative control, NC). (A) *Fmn1* mRNA expression in different experimental conditions. siRNA2 and siRNA3 were able to knockdown the expression of the gene while siRNA1 resulted ineffective. (B) Western blots showing the expression levels of Formin1 and β-Actin proteins after knockdown of *Fmn1* gene. Staining for GAPDH was included as loading control. Graph represents densitometric quantification of Western blots. Data show the mean ± SEM from three different experiments. Significance levels were determined using a Student t-test; * p<0.05, *** p<0.001.

To determine whether Formin1 mediates the effects of Ngn3 on neuronal morphology, three different double-stranded *Fmn1*-specific siRNA oligonucleotides were electroporated in hippocampal neuronal cultures. Figure 4A shows that two of the three siRNAs tested significantly reduced *Fmn1* mRNA expression. The Formin1 protein levels were also reduced after *Fmn1* gene knockdown, while no detectable β-actin level reduction was observed (Figure 4B), demonstrating the specificity of siRNA2 and siRNA3.

**Figure 5:**
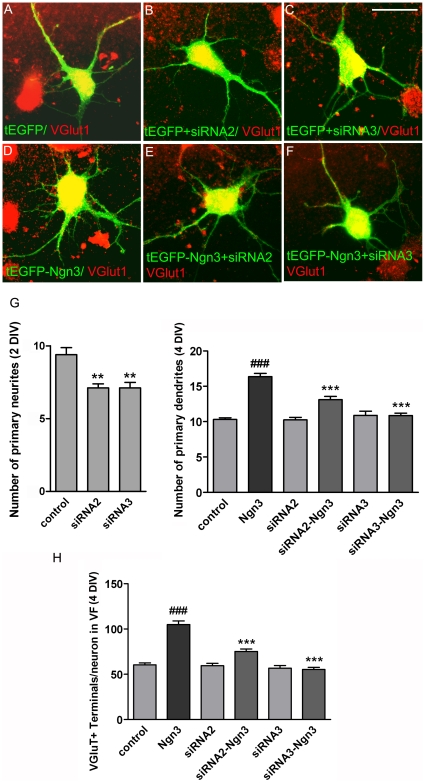
Effects of siRNAs targeting *Fmn1* on neurons overexpressing *Ngn3*. Hippocampal neuronal cultures were transfected at 1 or 3 DIV with pEGFP-C2 vector (control); co-transfected with either pEGFP-C2 plus *6xmyc-Ngn3*-expressing vector (Ngn3) or one of the siRNA oligonucleotides targeted to *Fmn1* (siRNA) or both (siRNA-Ngn3). After 16 h of expression time cultures were fixed and processed for immuncytochemistry for the analysis of neuritic morphology, dendritic morphology and glutamatergic synaptic inputs. (A–F) Representative immunofluorescence images of neurons marked in green for GFP and with VGlut1 synaptic terminals marked in red. Scale bar, 25 µm. (G) Number of primary neurites at 2 DIV and number of primary dendrites at 4 DIV. (H) Counts of VGlut1 immunoreactive terminals in contact with a neuron per ROI. ROI diameter: 50 µm. Data are mean ± SEM and significance levels were determined using a one way ANOVA followed by Bonferroni post hoc test, *** p<0.001 versus neurons overexpressing *Ngn3*; ### p<0.001 versus control neurons.

Next we co-transfected cells with siRNA oligonucleotides plus plasmid expressing *Ngn3* and *EGFP* or *EGFP* alone and assessed morphology and the number of glutamatergic presynaptic inputs in hippocampal neurons. Specimen images of transfected cells are presented in Figure 5, A–F. Morphometric evaluation of neurons shows that the addition, at 1 day in vitro (DIV), of *Fmn1*-specific siRNA oligonucleotides to cultures overexpressing *EGFP* alone induced a decrease in the number of neurites at 2 DIV (Figure 5G). To assess the synaptic input the transfection was made at 3 DIV and primary dendrites and glutamatergic presynaptic inputs were evaluated at 4 DIV. Under these conditions, overexpression of *Ngn3* induced an increase in the number of primary dendrites (Figure 5D and G) and in the number of glutamatergic synaptic terminals (Figure 5H). When *Fmn1*-specific siRNA2 and siRNA3 were added to cultures overexpressing *Ngn3* (Figure 5E and F), in both cases the effects induced by Ngn3 on primary dendrites and synaptic inputs were counteracted (Figure 5G and H), suggesting that Formin1 mediates the function of Ngn3 in the development of hippocampal neurons.

## Discussion

In the present study we used DNA microarrays to identify genes regulated by Ngn3 in cultured hippocampal neurons. Gene expression was compared between cultures overexpressing *Ngn3* and those overexpressing *GFP*. This comparative approach identified a set of genes, which are consistently regulated by Ngn3. Ngn3 regulates genes that are associated with cellular development, cellular growth and proliferation, cell morphology and cellular assembly and organization. Genes associated with the development and functioning of the nervous system are also regulated by Ngn3. Among the different groups of genes under the control of Ngn3, we focused on the genes related to cytoskeleton dynamics, since we were interested in elucidating the molecular mechanism by which Ngn3 controls morphology and synaptic inputs of hippocampal neurons [6]. One of those genes is *Fmn1*, which encodes a protein that nucleates actin [25] and associates with microtubules [20], [21]. The upregulation of *Fmn1* by Ngn3 was corroborated by direct assessment of gene expression at the mRNA and protein levels.

### The morphology and synaptogenesis of hippocampal neurons is regulated by *Fmn1*

The formation and maintenance of the neuronal dendritic tree depends on an underlying cytoskeleton consisting of a core of microtubules and a cortex of actin microfilaments [26]. Here we show that overexpression of *Fmn1-Ib* in cultured hippocampal neurons results in the expression of Formin1 in the neuronal cytoplasm and produces an increase in the number of primary dendrites. On the contrary the knocking down of the *Fmn1* gene induced a decrease in the number of neurites. These findings, together with the known effects of Formin1 on actin nucleation and microtubule association [21], [25], suggest that Formin1 participates in the control of neuronal morphology. Neuronal differentiation requires actin dynamics in the marginal growth cone region exhibiting actin-rich filopodia and lamellipodia. Filopodia contain bundled actin filaments and the lamellipodial veils contain a meshwork of actin filaments [27], [28]. Several actin assembly factors have been described to date and there are many studies aimed to understand their role during neurite growth. For example, the Arp2/3 complex promotes nucleation of a branched actin filament network in other cell types [29] and is implicated in axonal morphogenesis in several neuronal cell types [30]–[33]. Formins typically promote formation of unbranched actin filaments [34] and some members of the formin family are also involved in neuronal growth regulation. Thus, mDia1 has been linked to axonal elongation in cultured cerebellar granule cells [35], mDia2 is able to support filopodia formation, a prerequisite for neuritogenesis in cortical neurons [36] and the *Drosophila* formin Daam1 plays a critical role in axonal morphogenesis and induces the formation of neurite-like protrusions when expressed in mouse P19 cells [37]. Formin2, which is expressed in neurons throughout the central nervous system, has been shown to be transcriptional regulated in an age-dependent manner and functionally necessary for normal memory in mice [38]. Results from this study identify Formin1 as an actin assembly factor involved in the differentiation of hippocampal neurons.

Our findings also indicate that the number of glutamatergic presynaptic inputs is higher on neurons transfected with *EGFP-Fmn1-Ib* than on neurons transfected with *EGFP* alone. The increase in the number of glutamatergic presynaptic inputs may be, at least in part, a consequence of the generation of new dendritic spines in the postsynaptic neurons overexpressing *Fmn1-Ib*. Dendritic spines, small actin-rich protrusions from dendritic shafts, are the primary locus of excitatory synapses on neurons. Spine development starts with the initiation of the dendritic filopodium and its elongation followed by the spine head formation. A recent study has proposed that the mechanism of actin assembly is gradually changed from an mDia2-mediated polymerization of unbranched actin filaments to an Arp2/3-nucleated branched actin filament network, leading to enlargement of the spine head [39]. In addition over-expression of Daam1 decreases the density of dendritic spines and increases the spine length and area [40]. Our results suggest that Formin1 can also modulate actin-based synaptic structures.

### Loss of Formin1 function abrogates Ngn3 effects on neuronal morphology and synaptic input

We have demonstrated that *Ngn3* overexpression increases the number of primary dendrites and the number of excitatory glutamatergic synaptic inputs at 4DIV, in agreement with previous findings indicating that Ngn3 increases dendritogenesis and the ratio of excitatory/inhibitory synapses [6]. Here we demonstrate that these effects of Ngn3 are impaired by knocking down the *Fmn1* gene. However, the knocking down of the *Fmn1* gene at 1 DIV produced a decrease in the number of neurites in resting cultured hippocampal neurons but did not induce a significant effect on dendritogenesis and synapses at 3 DIV. This could be explained because at 3 DIV, Ngn3 levels are decreased compared to previous developmental stages [7] and the physiological effect of Ngn3 and Formin1 on dendritogenesis is difficult to observe. Thus, the fact that the knocking down of the *Fmn1* gene affects dendritogenesis and synapse number only in cells overexpressing Ngn3 suggests that Formin1 is needed for Ngn3-dependent initiation of new dendrites and synapses and not for the maintenance of previously generated ones. In conclusion, our findings indicate that Formin1 is involved in the Ngn3 signaling pathway that regulates neuronal morphology and synaptogenesis.

## Materials and Methods

### Ethics Statement

Mice were obtained from the Instituto Cajal and treated following the guidelines of Council of Europe Convention ETS123, recently revised as indicated in the Directive 86/609/EEC. In addition all protocols were approved by the Bioethics Committee of the “Consejo Superior de Investigaciones Científicas” (CSIC). Permit number: 28079/31A (01/08/2008).

### Hippocampal neuronal cultures

The hippocampus was dissected out from embryonic day 17 CD1 mouse embryos and dissociated to single cells after digestion with trypsin (Worthington Biochemicals, Freehold, NJ) and DNase I (Sigma-Aldrich) [41]. Neurons were plated on 6-wells plates or glass coverslips coated with poly-L-lysine (Sigma-Aldrich) at a density of 150–300 neurons/mm^2^, and they were cultured in Neurobasal supplemented with B-27 and GlutaMAX I (Invitrogen, Crewe, United Kingdom). Under the conditions used, our cultures were nearly devoid of glia.

### Transfection

Neurons were transfected at 1 and 3 DIV using the Effectene Transfection Reagent (Qiagen GmbH, Hilden, Germany), following the manufacturer's instructions. Cells were either transfected with an *EGFP-* or *EGFP-Fmn1-Ib*-expressing *C2* vector [21]; Clontech, USA) or co-transfected with *pEGFP-C2* plus *6xmyc-Ngn3*-expressing *CS2+* vector or one of the siRNA oligonucleotides targeted to *Fmn1*. After 16h of expression time the cultures were fixed in 4% paraformaldehyde in 0.1 M phosphate buffer for immunostaining.

### Nucleofection

The same plasmids and small interfering RNAs (siRNAs) targeted to *Fmn1* or *Ngn3* were nucleofected into cultured neurons using an Amaxa nucleofector with the Mouse Neuron Kit (Amaxa, Gaithersburg, MD) according to the manufacturer's instructions and after 1–3 DIV, the neurons were harvested and processed to real time PCR or to Western blotting analysis.

### SiRNA

The siRNA oligonucleotides were purchased from Applied Biosystems/Ambion and the concentration was 30 nM during transfection. SiRNAs targeting *Ngn3* were: *Ngn3*-siRNA1 (sense, AACUACAUCUGGGCACUGAtt; antisense, UCAGUGCCCAGAUGUAGUUgt); *Ngn3*-siRNA2 (sense, GCUUCUCAUCGGUACCCUUtt; antisense, AAGGGUACCGAUGAGAAGCct) whose extent and specificity of gene silencing has been describe elsewhere [42].

SiRNAs targeting *Fmn1* were: *Fmn1*-siRNA1 (sense, GGAUGAACUGACUAAAAUAtt; antisense, UAUUUUAGUCAGUUCAUCCtc); *Fmn1*-siRNA2 (sense, GGCGACAUAUUUUUCAAACtt; antisense, GUUUGAAAAAUAUGUCGCCtg) and *Fmn1*-siRNA3 (sense, CCUUUGUAUUGGACCAGAAtt; antisense, UUCUGGUCCAAUACAAAGGtt). Control included non targeting siRNA (NC). The extent and specificity of *Fmn1* gene silencing were assessed by real time RT-PCR and by Western blotting.

### Microarray gene expression data and analysis

Hippocampal neurons were transduced at 5 DIV using Sindbis virus bearing *myc-tagged Ngn3*, prepared as described before [7] or *GFP* as control. After 1 h viral particles were removed and proteins were allowed to express during 16 h. Next cells were lysed and total RNA was extracted using illustra RNAspin Mini RNA isolation kit from GE Healthcare (Buckinghamshire, UK). RNA quality was analyzed using a BioAnalyzer (Agilent Technologies, Santa Clara, CA). Differential gene expression analysis between the experimental populations (neurons expressing *6xmyc-Ngn3*) and the corresponding control populations (neurons expressing *GFP*) was performed using one-color CodeLink Whole Mouse Genome Bioarrays (GE/Amersham, Piscataway, NJ, now Applied Microarrays, Tempe, AZ) according to manufacturer's recommendations. In order to obtain results with statistical significance, four biological replicates were analyzed per experimental group; therefore 8 RNA samples were analyzed. Hybridized arrays were scanned on an Agilent Microarray Scanner (G2565BA, Agilent Technologies) and CodeLink Expression Analysis software was used for primary data extraction from bioarray images. All data from the DNA microarray analyses are MIAME compliant and have been deposited in the Gene Expression Omnibus (GEO) data repository under the GEO accession number [GEO: GSE26911]. Microarray data were analyzed using the R language and packages from the Bioconductor project (http://www.bioconductor.org/). The *codelink* [43] package was used for preprocessing the arrays, *genefilter* [44] for data filtering and *limma* [45] for statistical analysis. For preprocessing, background was corrected using the *normexp* method and *quantile* normalization was performed. Data were considered in the log2 scale. P-values were adjusted to control the False Discovery Rate (FDR) using the Benjamini and Hochberg correction [46]. Genes with a Benjamini-Hochberg adjusted p-value smaller than 0.05 were selected as differentially expressed.

Functional analysis was performed using Ingenuity Pathway Analysis (Ingenuity Systems®, www.ingenuity.com). This analysis identified the functions and/or diseases that were most significant to the dataset. Genes from the dataset that were associated with biological functions and/or diseases in the Ingenuity knowledge base were considered for the analysis. Fischer's exact test was used to calculate a p-value determining the probability that each biological function and/or disease assigned to the data set is due to chance alone.

### Quantitative real-time polymerase chain reaction (PCR)

First strand cDNA was prepared from RNA using the First Strand Synthesis kit from Fermentas GMBH (St Leon-Rot, Germany) following the manufacturer instructions. Quantitative real-time PCR was performed using the ABI Prism 7000 Sequence Detector (Applied Biosystems). TaqMan probes and primers for *Ngn3*, *Fmn1* and for the control housekeeping gene, *GAPDH*, were Assay-on-Demand gene expression products (Applied Biosystems). Real-time PCRs were performed following the suppliers instructions using the TaqMan Universal PCR Master Mix. All reactions were done in triplicates, from 3 different cultures. *Ngn3* and *Fmn1* expression were normalized for *GAPDH* expression. The data were analyzed with an unpaired t-test or one-way analysis of variance (ANOVA) followed by the Bonferroni post hoc test using GraphPad Prism 5 (GraphPad Software, Inc., San Diego, CA).

### Antibodies

The following primary antibodies were used: goat anti-Formin1 (1∶200; Santa Cruz Biotechnology, Inc.); mouse anti-myc (1∶200; Roche, Indianapolis, USA); chicken anti-GFP (1∶1000; Abcam, Cambridge, UK); guinea pig anti-vesicular glutamate transporter 1 (VGlut1) (1∶500; Millipore Corporation); rabbit anti-vesicular GABA transporter (VGAT) (1∶250; Millipore Corporation); mouse anti-GAPDH (1∶500, Millipore Corporation) and mouse anti-β-actin (1∶5000, Sigma). All secondary antibodies were from Jackson Immuno Research (West Grove, PA, USA).

### Western Blotting

Proteins were resolved by SDS-PAGE and transferred onto polyvinylidene difluoride membranes (Millipore). The membranes were blocked in Tris-buffered saline containing 0.3% Tween 20 and 5% fat-free dry milk and incubated first with primary antibodies and then with horseradish peroxidase-conjugated secondary antibodies. Specific proteins were visualized with enhanced chemiluminescence detection reagent according to the manufacturer's instructions (Amersham). Densitometry and quantification of the bands were carried out using the Quantity One software (Bio-Rad). Statistical analysis of the data was performed using an unpaired t-test.

### Image acquisition and analysis of labeled hippocampal neurons

Images were acquired digitally using a 20× or 40× oil immersion objective and fluorescence filters in a Leica (Bensheim, Germany) microscope. Photomicrographs were stored and digitally processed with Adobe Photoshop, v. 7.0 (Adobe Systems, San Jose, CA). Only minor adjustments to brightness and contrast were made. Primary neurite number at 2 DIV and primary dendrite number at 4 DIV (i.e., the number of neurites or dendrites emerging from the soma) and synaptic terminal counts at 4 DIV were performed manually. A circular region of interest (ROI) with a diameter of 100 µm was projected onto the GFP labeled neuron, its center roughly coinciding with the center of the soma. Synaptic terminals contacting somata or dendrites were counted within the circular ROI.

## Supporting Information

Table S1 **Differentially Expressed Genes (Control versus** ***Ngn3*** **overexpresed).** Shown are the genes selected as differentially expressed (adjusted p-value smaller than 0.05) and the fold changes (represented in log2 scale). The sign indicates the direction of the change: positive values refer to greater transcript abundance in the control cultures, whereas negative values indicate less abundance in the controls (and higher abundance in the *Ngn3* overexpressing neurons).
(XLS)

Table S2 **Functional analysis of differentially expressed genes in Ngn3 overexpressing neurons.** Genes are classified according to their known biological function.
(XLS)
